# Comparative Evaluation of Three Phenotypic Tests—Carba NP, Modified Carba NP and Rapidec Carba NP Test for Rapid Detection of Carbapenem Resistance in Blood Culture Isolates of *Escherichia coli* in an ICU Setting

**DOI:** 10.21315/mjms2022.29.6.6

**Published:** 2022-12-22

**Authors:** Ivneet Kour, Dipanshu Vasesi, Lipika Singhal, Varsha Gupta

**Affiliations:** Departments of Microbiology, Government Medical College and Hospital, Chandigarh, India

**Keywords:** Carba NP, carbapenem resistance, extended spectrum beta lactamases, Modified Carba NP

## Abstract

**Background:**

To determine the antibiotic resistance pattern, the prevalence of extended-spectrum beta-lactamases (ESBLs) and carbapenem resistance in blood culture isolates of *E. coli*. Further, we evaluated and compared Carba NP, Modified Carba NP and a kit-based Rapidec Carba NP test to detect carbapenem resistance rapidly.

**Methods:**

Twenty-six carbapenem-resistant strains and four susceptible strains were selected. The three methods mentioned above were evaluated as per Clinical Laboratory Standards Institute (CLSI). These tests are based on biochemical detection of the hydrolysis of the beta-lactam ring of a carbapenem-imipenem, followed by the colour change of a pH indicator.

**Results:**

Carba NP test was positive in 24 out of 26 isolates; the Modified Carba NP and Rapidec Carba NP tests were positive for all the isolates (26/26). All the carbapenemase non-producers (100%, 04/04) were negative.

**Conclusion:**

Modified Carba NP is a more effortless and inexpensive alternative to the Carba NP test, allowing the detection of carbapenemase activity directly from bacterial cultures of *Enterobacteriaceae*. The test could be used in low-income countries with large reservoirs for carbapenemase producers and can be implemented in any laboratory worldwide.

## Introduction

Bloodstream infections (BSIs) are among the most common community and hospital-acquired infections worldwide and are severe public health problems. BSIs are common in intensive care units (ICUs) and have been shown to predict severe sepsis. Community-acquired BSIs, which are usually caused by susceptible bacteria, should be distinguished from hospital-acquired BSIs, which are commonly caused by resistant hospital strains (1). Gram-negative bacteria are responsible for approximately 25% of all occurrences of healthcare-associated bacteraemia and almost 50% of all cases of community-acquired bacteraemia. They enter the bloodstream most commonly through infections in the respiratory tract, genitourinary tract, gastrointestinal tract or hepatobiliary system (2). Gram-negative bacteraemia is more common in elderly people (60 years old plus of age) and is linked to a greater rate of morbidity. It has been stated that multidrug resistant (MDR) Gram-negative bacteria associated with bacteraemia commonly were *Escherichia coli, Klebsiella pneumoniae, Proteus mirabilis* and *K. oxytoca* (3).

*E. coli* is frequently isolated in adult patients with bacteraemia (4). Antibiotics are commonly prescribed everywhere as a part of both empirical and regular therapy for BSIs. Multiple resistance mechanisms have been reported increasingly in *E. coli* isolates, one of which is the phenotypic expression of plasmid-mediated genes encoding extended-spectrum beta-lactamases (ESBL). ESBLs are Ambler class A or D β-lactamases that confer resistance to most beta-lactam antibiotics, including 3rd and 4th generation cephalosporins and monobactams (5). Carbapenems are recommended as the last-resort antibiotics for treating infections with ESBL-producing *E. coli* (6). However, the increased use of carbapenems has led to the emergence of carbapenem-resistant strains referred to as carbapenem-resistant *Enterobacteriaceae (CRE) (*7).

A variety of carbapenem-hydrolysing beta-lactamases (carbapenemases) have been reported in *Enterobacteriaceae*, such as KPC (Ambler class A), metallo beta-lactamases of VIM-, IMP- and NDM-type (Ambler class B) and OXA-48-types (Ambler class D). These carbapenemase genes are more diverse and laboratory detection is more challenging (7, 8). This poses a significant threat to hospitalised patients and in the community setting. Thus, an efficient strategy for detecting carbapenem resistance is essential to determine the appropriate therapeutic modalities.

As India is one of the largest consumers of antibiotics globally, the efficacy of several antibiotics is compromised due to the emergence of resistant bacterial strains. Antimicrobial resistance threatens healthcare at every level and has become a major international concern for public health. Bacterial pathogens can evolve to transmit, cause disease and resist antibiotics. As bacteria evolve with an increased risk to human health, we need systematic approaches for collecting and identifying these at a local level and integrated systems to collate data to provide an international overview. In this study, we aimed to determine the antibiotic resistance pattern, prevalence of ESBL’s and carbapenem resistance in blood culture isolates of *E. coli* in an ICU setting. We further evaluated and compared the results of Carba NP, Modified Carba NP and a commercially available kit-based Rapidec Carba NP test (BioMérieux SA) for rapid detection of carbapenem resistance.

## Methods

This prospective study was conducted in the Department of Microbiology of a tertiary care hospital in North India on the *E. coli* strains isolated from blood samples collected from ICUs after following all aseptic precautions. In total, 68 non-duplicate carbapenem-resistant clinical isolates of *E. coli* were included in the study. The bacterial isolates were identified to species level as per standard microbiological procedures, which were collected for a period of 1 year (2019–2020). Based on Clinical Laboratory Standards Institute (CLSI) 2021 guidelines, the antimicrobial susceptibility of the following drugs was determined by the Kirby-Bauer method: amikacin (AMK, 30 μg), ciprofloxacin (CIP, 5 μg), piperacillin-tazobactam (PTZ, 100/10 μg), cefotaxime (CTZ, 30 μg), ceftazidime (CAZ, 30 μg), cefepime (CEP, 30 μg), imipenem (IMP, 10 μg)/meropenem (MEM, 30 μg). ESBL production was detected by the CLSI method using CAZ and CAZ-clavulanic acid combination disks (9). Further confirmation of ESBL non-producers was performed using boronic acid (BA) as an inhibitor (10). Carbapenem-resistant strains were detected by using ertapenem (10 μg)/imipenem (10 μg)/meropenem (10 μg) (BD, Diagnostics) discs and the resistant strains were confirmed by minimum inhibitory concentration (MIC) determination using meropenem E-test (bioMerieux India Pvt. Ltd.). Carbapenem resistance was reported if MIC to meropenem was ≥ 4 μg/mL. Standard strains of *E. coli* (ATCC 25922) were used as a control.

The detected carbapenem-resistant strains were then tested by the Carba NP, Modified Carba NP, and Rapidec Carba NP Kit tests and the results were evaluated. These tests are based on biochemical detection of the hydrolysis of the beta-lactam ring of a carbapenem-imipenem, followed by the colour change of a pH indicator (11, 12). Carba NP test uses reference standard imipenem powder, while Modified Carba NP uses a therapeutic IV imipenem/cilastatin. Rapidec Carba NP kit is a ready-to-use commercial kit (BioMérieux). These tests were performed on strains grown on Mueller-Hinton agar plates in triplicates for each isolate and results were interpreted by more than one independent reader (13, 14).

According to the protocol, all isolates were then subjected to the Rapidec Carba NP test. A 10-μL loop was used to pick up a bacterial colony from overnight-incubated MHA plates (BioMérieux) and then mixed into a kit-specific API suspension medium. The bacterial suspension was then transferred to wells in the test strip and incubation at 37 °C. The test strip was read visually after 30 min and 2 h. A colour change from red to yellow-orange was considered positive, whereas red was interpreted as a negative result (15).

Four non-carbapenemase producers, of which none was resistant to any of the carbapenems, were included in the study. In this group, the Modified Hodge Test (MHT) was negative and we concluded that all other mechanisms of carbapenem resistance like overexpression of chromosomal AmpC or expression of plasmid-mediated AmpC and/or ESBLs coupled to impermeability or efflux pumps were not there (16). Quality control strains used were *K. pneumoniae* ATCC BAA-1705-MHT positive and *K. pneumoniae* ATCC BAA-1706-MHT negative.

## Statistical Analysis

In this study, Statistical Package of Social Science (SPSS) version 22.0 software was used for calculating the indices like sensitivity and specificity of Carba NP, Modified Carba NP and Rapidec Carba NP test for rapid detection of carbapenem resistance.

## Results

**Figure 1:**
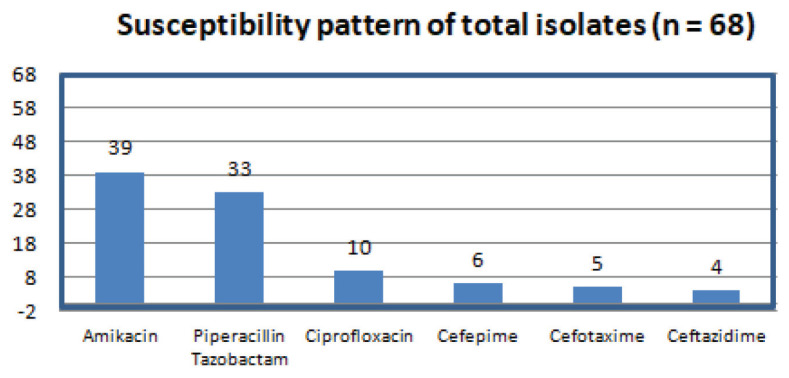
The antibiogram pattern of isolates of *E. coli*

Out of the total 68 strains of *E. coli*, which were isolated from blood culture samples of patients in an ICU setting, 26 isolates were carbapenem-resistant and were therefore selected for the study. The antibiotic susceptibility profiles of 68 isolates were analysed (Figure 1). The most effective antibiotics for these isolates were fluroqunilones and piperacilin-tazobactam with susceptibilities being 60% (39/68) and 51% (33/68), respectively as shown in Table 1. Piperacilin belongs to penicilin class of antibiotics and tazobactam is a beta-lactamase inhibitor. The prevalence of ESBL positive strains amongst the 68 strains of *E. coli* were 36 strains (53%). The prevalence of carbapenem resistance was observed in 26 isolates out of the 68 strains (38% resistance).

Amongst the 26 carbapenem-resistant strains of *E. coli*, the number of ESBL positive strains was 21 (80%) and ESBL negative strains were only 5 (20%). These strains were resistant to imepenem, ciprofloxacin, cefepime, cefotaxime and ceftazidime. Based on carbapenemase detection conducted on these carbapenem-resistant isolates, MHT was positive in 18 of these 26 isolates. Carba NP test was positive on 24/26, Modified Carba NP and the kit based Rapidec Carba NP test were positive for all the isolates (26/26). The sensitivity varied with Modified Carba NP and the kit-based Rapidec Carba NP test performed slightly better than the Carba NP test. All the four carbapenemase non-producers included in the study were negative by these three tests (4/4, 100%). These carbapenem susceptible strains were tested to rule out or minimise false positivity and evaluate the test methodology’s specificity. The specificities of all three tests were excellent.

## Discussion

Although highly resistant strains are yet not widespread in countries with strict antibiotic regimes, multi-drug resistance and ESBL resistance are frequently detected in *E. coli* isolated in blood culture. As far as the prevalence of ESBLs among clinical isolates is concerned, it varies around the world, being low in countries like the United States and Canada (6%–11%) and high in others such as India where antibiotic usage is not as controlled (17).

Based on studies done in the last two decades, India has reported vast variations over geographical zones and with time. ESBL production rates in Gram-negative bacilli isolated from blood culture differ from 50% to 87% (5, 18, 19).

A recent study in North India reported that 33% of clinical isolates of *E. coli* produced ESBLs (20). However, in the present study we found that a shockingly high percentage of isolates (53%) produced ESBLs as reported by Maya et al. (75%) (21).

The reason for such wide variations may be due to the antibiotic susceptibility of pathogens shows high inter-regional variation and that is associated with clinical practices of physicians and medications of patients as well as evolutionary resistance resulting in certain pathogens becoming more resistant over time. The production of ESBLs by the isolate limits treatment choice because ESBL production is often associated with co-resistance to other antimicrobial agent classes like fluoroquinolones and aminoglycosides. This alarming increase in resistance possesses a therapeutic challenge that can exacerbate the severity of even mild sepsis.

Regarding the antibiotic susceptibility, *E. coli* was found to be vulnerable to a beta-lactamase/beta-lactamase inhibitor (BL/BLI) combination such as piperacillin-tazobactam, aminoglycosides and carbapenems. However, all of these are parenteral antibiotics with a limited usage for indoor patients. Carbapenems should be reserved for severe cases and a carbapenem sparing policy should be implemented to stop emergence of resistance. In outpatient setting, oral antibiotics are always preferred although very limited options are left for sepsis treatment. The oral drugs such asciprofloxacin and amoxycillin-clavulanate can be used, but only after susceptibility reports are available as empirical therapy which may not be helpful in the present scenario. Moreover, the choice of antibiotic must be based on several aspects like clinical condition, renal function tests and whether the patient is indoors or outside.

The present study also highlights the supreme importance of detection of carbapenem resistance for effective therapy. The Carba NP test is the most important and recent development for the accurate identification of carbapenemase producing *Enterobacteriaceae* (22). The Carba NP test is a novel phenotypic method for carbapenemase detection. It is based on the in vitro hydrolysis of imipenem by a bacterial lysate, which is detected by changes in pH values by using the indicator phenol red (red to yellow/orange) (13).

The Modified Carba NP test is another variant that uses 0.02% cetyltrimethylammonium bromide lysis buffer and a starting pH of 7.5 instead of 7.8, allowing for better carbapenemase producer identification. It also detects OXA 48 carbapenemases to some extent (14).

Hydrolysis of imipenem is detected by a change in the pH value of the indicator (from red to yellow/orange). These tests are rapid (2 h), easy to use and does not require any specific equipment. Various studies have reported this test to be 100% sensitive and specific, according to the molecular techniques (11, 20, 21). It detects not only all known carbapenemases (belonging to Ambler A, B and D classes) in *Enterobacteriaceae*, but also identify virtually any new emerging carbapenemase in contrast to molecular techniques. MHT was performed for carbapenem detection, while Carba NP showed better results. However, we found a lower sensitivity as it identified 24/26 isolates (92%) correctly on multiple occasions with no false positives (100% specificity). Due to the high cost of reference standard imipenem powder and its instability in solution form, this test is also costly, labour-intensive and inconvenient. To identify a cheaper or more convenient test of similar accuracy, a Modified Carba NP test that used intravenous imipenem/cilastatin which is cheaper and more stable than the reference standard imipenem powder (Primpen). Along with this modification, a ready-to-use commercial Rapidec Carba NP kit obtained from BioMerieux was also evaluated. Our Carba NP test was modified and provided positive findings with 100% sensitivity and specificity. The commercial kit performed equally well but it is too expensive to be used in routine laboratories of a resource-limited nation like ours.

A second experiment was conducted by using more cells to evaluate a more concentrated extract. In this case, the Carba NP negative and Modified Carba NP positive isolates were retested by using a more concentrated extract. When the inoculum was higher, the Carba NP test showed clearer colour changes that were positive. Similar findings have been documented in a study by Tijet et al. (23), suggesting OXA 48 could be the reason. These results improved the overall sensitivity of the Carba NP test in our study. The limitation of our study was that we were unable to perform molecular based identification of the various carbapenemases and confirm the reason for the discrepant result.

## Conclusion

The Modified Carba NP is an easier and cheaper alternative to the Carba NP test, allowing carbapenemase activity to be deleted directly from bacterial cultures of *Enterobacteriaceae*. The test can be used in low-income countries that have large reservoirs for carbapenemase producers and can be implemented in any laboratory worldwide. It offers a practical solution for detecting the primary component of multidrug resistance in *Enterobacteriaceae*. These tests have the potential to contribute to a better stewardship of carbapenems by changing the paradigm of controlling carbapenemase producers worldwide, especially in ICU patients. Further, the best choice of empirical therapy option should be determined. The aetiology agents of BSI should be monitored periodically and their resistance patterns as well. This study would help to explore the possibilities for revising the antimicrobial stewardship programme which would reduce morbidity and hospitalisation costs.
